# Evidence-based practice and clinical practice in child language: an online survey with Brazilian speech-language pathologists

**DOI:** 10.1590/2317-1782/20232022272en

**Published:** 2023-11-10

**Authors:** Marcelle Stella de Lima Souza, Ana Manhani Cáceres-Assenço

**Affiliations:** 1 Laboratório de Desenvolvimento da Linguagem, Departamento de Fonoaudiologia, Centro de Ciências da Saúde, Universidade Federal do Rio Grande do Norte - UFRN - Natal (RN), Brasil.

**Keywords:** Child Language, Specific Language Disorder, Evidence-Based Practice, Language Therapy, Rehabilitation of Speech and Language Disorders

## Abstract

**Purpose:**

To characterize the knowledge, skills, opinions, and main barriers perceived by speech-language pathologists, in child language in Brazil, regarding evidence-based practice (EBP).

**Methods:**

The study was conducted between August 2021 and July 2022 using an online questionnaire. In addition to sociodemographic and field data, 22 items related to EBP were considered and subdivided into “knowledge”, “skills”, “opinion” and “barriers”. Each item had five response options (strongly disagree, disagree, not decided, agree, strongly agree). A total of 122 speech-language pathologists who work with child language answered the questionnaire. Their responses were described by the percentage of frequency distribution. The time since graduation and the level of proficiency in English were considered to compare the distribution pattern of the answers.

**Results:**

Although most speech-language pathologists report having learned the basics of EBP in their academic training, there are weaknesses in their knowledge and lack of mastery of search strategies and critical evaluation of scientific articles. Although most agree that EBP’s use is necessary for speech-language practice and consider the need to increase the use of scientific evidence in their daily practice, the lack of articles, difficulties related to the practical application of scientific results and lack of collective support among colleagues are identified as barriers.

**Conclusion:**

This study alerts the academic community to the urgency of considering EBP in the context of Brazilian Speech-Language Pathology.

## INTRODUCTION

Evidence-based practice (EBP) is a set of criteria for evaluating scientific evidence. The main objective of EBP is to reduce the uncertainty of the professional at the time of a clinical decision. It associates three pillars: clinical experience of the professional; preferences of the family and/or the client; and external (information available in the literature) and internal evidence (data collected in the evaluation)^(1,2)^.

The importance of EBP has been frequently discussed in the medical and scientific community^(3)^. However, there are still many barriers that prevent its effective implementation, especially in Speech-Language Pathology^(4-6)^. In summary, despite having some theoretical basis, speech-language pathologists (SLP) who work with language disorders in the international scenario recognize the insufficient time, the extensive workload, the scarcity of research in the area, the quality of available evidence and the lack of resources in the work environment as the main obstacles to the implementation of EBP^(4,5,7,8)^.

Scientific evidence does not seem to be decisive for the selection of intervention approaches, especially in the performance with child language. The most considered factor for decision-making is the clinical experience of the SLP. As much as the professional's experience is relevant, the effectiveness of EBP depends on its association with internal evidence and client preferences^(9,10)^. However, it is essential to point out that in language disorders studies with the best levels of evidence are still scarce and there is a deficit in the knowledge of professionals regarding the processes of diagnosis and speech-language intervention^(9,11)^.

In an international context, SLPs who work with language, in general, have positive attitudes and are favorable to the implementation of EBP, although there are still barriers^(4,5,7,8)^. Formal training on EBP at graduation or during continuing education appears as a strong predictor for the execution of such practice during its clinical performance^(4,6,12)^.

However, in the Brazilian context, there is a lack of studies that investigate such a scenario. Therefore, the aim of the study was to characterize the knowledge, skills, opinions, and main barriers perceived by SLPs in child language in Brazil, regarding evidence-based practice.

## METHODS

This study is linked to a broader project that investigates how Brazilian SLPs act in the diagnosis and intervention in child language. The project was approved by the Research Ethics Committee (nº 4,878,557). The informed consent form was presented before the questionnaire and participation was voluntary and anonymous. The participants were informed about the project’s objectives, the estimated time for response and how to contact the researchers in case of questions. The guidelines of the National Research Ethics Commission (CONEP) for procedures in research in a virtual environment, published in February 2021, were followed in order to preserve the protection, safety and rights of participants.

### Materials and procedure

The first stage of the study consisted of the elaboration of a questionnaire on evidence-based practice from instruments used by Physical Therapy^(12,13)^. The questionnaire was composed of 22 items subdivided into the categories “knowledge”, “skills”, “opinion” and “barriers”. In each item it was necessary to specify the level of agreement by a Likert scale with five response options (strongly disagree, disagree, not decided, agree, strongly agree). The questionnaire was preceded by questions related to sociodemographic aspects and work field.

The questionnaire was available in an open form on the platform *Google Forms*. Before data collection began, we asked five undergraduate students to complete the questionnaire in search of errors or inconsistencies, aiming to improve the applicability of the instrument.

Contact with potential participants occurred by social media. Instagram mentions of digital influencers and a sponsored ad on Instagram targeted SLPs with an interest in child language, the study was sent by email in the research newsletter to the associates of the Brazilian Society of Speech-Language Pathology (SBFa). Due to the recent implementation of the general data protection law, the Federal Council and the Regional Council of Speech-Language Pathology reported that it was unfeasible to disseminate the study by e-mail to active professionals.

Access to the questionnaire was provided by a shortened link. All questions were mandatory, and the order of presentation was standardized. The questionnaire was spread over eight pages, each with about five multiple-choice questions. When submitting the questionnaire, it was no longer possible to make changes and a copy of the answers was sent to the participant. The answers were automatically stored in a Google spreadsheet.

Due to the characteristics of the platform *Google Forms* survey view rates could not be calculated. However, all questionnaires submitted indicated agreement with the study and were complete. A priori, no measures were adopted to prevent duplication of responses, however e-mail was used to eliminate these occurrences before the analysis. In this sample we had three duplicate forms and chose to keep the first submission.

### Participants

As inclusion criteria, participants should have a degree in Speech-Language Pathology and work with language disorders in childhood. According to the Federal Council of Speech-Language Pathology, in June 2021 there were 48,391 SLPs in Brazil. Of these, 1155 hold the title of language specialist. However, not every professional who works with child language has the title of specialist and not every specialist works with child language. Thus, it was not possible to have an accurate estimate of the population of interest.

The sample calculation was performed using GPower software. For an effect size of 0.3 and a statistical power of 0.8, the estimated sample would be 167 participants. In order to reach this sample, data collection took place between August 2021 and July 2022.

Finally, 122 SLPs participated in this study who declared to act clinically in the area of child language in Brazil. The group was composed of 96.7% by women, with the predominant age group between 41 and 50 years (32.8%), university education in a private institution (53.3%) and for less than 5 years (31.1%), with specialization in the area (36.1%), according to Table 1.

**Table 1:** Frequency distribution of sample characterization

Characteristics	%
Gender	
Female	96.7
Male	3.3
Age	
20-30	24.6
31-40	29.5
41-50	32.8
51-60	10.7
+61	2.5
Type of institution you graduated from	
Private	53.3
Public	46.7
Training time	
- 5 years	31.1
5 to 9 years	18.0
10 to 14 years	13.1
15 to 19 years old	9.8
20 to 24 years	17.2
+24 years	10.7
Level of professional qualification	
Doctorate	12.3
Specialization *lato sensu*	36.1
Graduation	23.8
Academic masters	19.7
Professional masters	4.1
Postdoctoral	4.1

### Data analysis

The statistical treatment of the data was performed in the SPSS software version 24. Frequency distribution was used for descriptive analysis. The inferential analysis was performed using the chi-square test considering for the time since graduation two categories (up to 9 years since graduation and from 10 years since graduation) and for English proficiency three categories (poor, moderate, and good or excellent). The significance level adopted was 5%. In addition to the sample calculation, the software *GPower* was used to calculate effect size and statistical power.

## RESULTS

Participants expressed concern about continuing education, as 82% claim to have participated in scientific congresses, courses or updates in the area (40.2% very often, 41.8% often). In addition, 63.1% claim to have the habit of reading scientific articles, 20.5% very often and 42.6% often. The databases most used for searching articles were Scielo (33.6%), PubMed (24.8%) and Google Scholar (23.2%). None of the participants indicated using the SpeechBite Database (Table 2).

**Table 2:** Frequency distribution of the search for continuing education and external evidence

Characteristics	%
Continuing education in the last 3 years	
Very often	40.2
Frequently	41.8
Occasionally	13.9
Rarely	2.5
Never	1.6
Reading articles	
Very often	20.5
Frequently	42.6
Occasionally	29.5
Rarely	6.6
Never	0.8
Database used	
Scielo	33.6
PubMed	24.8
Google Scholar	23.2
Capes Newspapers	8.8
ASHA evidence map	6.4
Not applicable	3.2
Cochrane	0.8
Bireme	0.8
SpeechBite	0.0

### Knowledge

Regarding knowledge, most participants indicated that they had learned the basics of EBP during their academic training (36.1% agree and 20.5% strongly agree) and consider that it improves the quality of care (35.2% agree and 54.1% strongly agree) and helps in making decisions about treatment (47.5% agree and 39.3% strongly agree). However, inconsistencies were identified in the items. “*PBE does not take into account the limitations of my clinical practice*” (45.1% not decided, 19.7% agree and 3.3% strongly agree) and “*EBP does not take into account patient preferences*” (29.5% are not decided, 15.6% agree and 2.5% strongly agree), according to Table 3.

**Table 3:** Barriers, skills, knowledge and opinion of speech therapists on evidence-based practice

	Strongly disagree	Disagree	Not decided	Agree	Strongly agree	X^2^ English Mastery	X^2^ Time since graduation
**Knowledge**							
In my academic training I learned the basics for EBP	12.3	15.6	15.6	36.1	20.5	<0.001*	0.021*
EBP improves the quality of patient care	0.0	0.8	9.8	35.2	54.1	0.314	0.059
EBP does not take into account the limitations of my clinical practice	6.6	25.4	45.1	19.7	3.3	0.203	0.290
EBP does not take into account patient preferences	10.7	41.8	29.5	15.6	2.5	0.197	0.343
EBP helps make decisions about patients' treatment	0.8	1.6	10.7	47.5	39.3	0.053	0.584
**Skills**							
I use EBP in clinical practice and therapeutic planning	1.6	2.5	10.7	44.3	41.0	0.018*	0.266
I am interested in learning or enhancing the skills needed to incorporate EBP into my practice	0.0	0.0	2.5	37.7	59.8	0.521	0.106
I have training in search strategies to find online literature relevant to my practice	9.0	21.3	14.8	30.3	24.6	0,001*	0.181
I have formal training in critical evaluation of scientific articles as part of my academic preparation	15.6	22.1	17.2	19.7	25.4	0.003*	0.172
I am able to do a critical analysis of scientific papers	0.8	14.8	21.3	38.5	24.6	<0.001*	0.204
I am able to find relevant scientific articles to answer my clinical questions	0.8	8.2	18.0	43.4	29.5	<0.001*	0.421
I am able to understand the statistical analysis of scientific papers	4.9	12.3	31.1	31.1	20.5	<0.001*	0.173
**Opinion**							
In my perspective, the application of EBP is necessary for the practice of Speech Therapy	0.0	0.0	7.4	32.8	59.8	0.156	0.419
I need to increase the use of scientific evidence in my daily practice.	1.6	12.3	7.4	42.6	36.1	0.348	0.103
My financial gain will increase if I incorporate EBP into my practice	11.5	23.0	33.6	26.2	5.7	0.872	0.285
Strong scientific evidence is lacking for most of the interventions I use on patients	8.2	35.2	28.7	22.1	5.7	0.239	0.046
The incorporation of EBP places too much responsibility on the speech therapist	14.8	34.4	26.2	19.7	4.9	0.009*	0.097
**Barriers**							
The place where I work encourages the use of the results of current investigations in clinical practice	10.7	14.8	15.6	30.3	28.7	0.220	0.290
The time available is insufficient for the implementation of the EBP	5.7	31.1	35.2	23.0	4.9	0.537	0.036
There is a lack of articles that make it possible to generalize the findings of the scientific literature to my patient population	2.5	20.5	21.3	41.0	14.8	0.469	0.399
There is difficulty in applying the results of scientific research to patients with unique characteristics	0.8	16.4	26.2	43.4	13.1	0.653	0.679
There is a lack of collective support among my co-workers for the implementation of EBP	5.7	20.5	25.4	37.7	10.7	0.747	0.880

* Statistical difference p<0.05-chi-square test (X^2^)

The inferential analysis indicated differences according to English proficiency (p<0.001) and time since graduation (p=0.021) only in the item “*In my academic training I learned the basics for EBP*”. With regard to English proficiency, the difference suggests that SLPs with poor proficiency have less knowledge about EBP than those with moderate or good and excellent mastery. Regarding training time, the difference suggests that SLPs up to 9 years after graduation were less decided about having had access to this content than their peers trained more than 10 years ago.

### Skills

With regard to skills, most participants indicated using EBP (44.3% agree and 41.0% strongly agree) and having an interest in learning or improving their skills (37.3% agree and 59.8% strongly agree). However, when it comes to formal training in critical evaluation of scientific articles, most disagree or are neutral (15.6% disagree, 22.1% strongly disagree, 17.2% not decided).

24.6% of SLPs indicated that they had training in strategies for searching for scientific articles, while 22.1% of them disagreed with this statement. In addition, 31.1% consider themselves capable of understanding the statistical analysis of the articles, but another 31.1% were not decided about this item, as shown in Table 3.

Inferential analysis indicated differences according to English proficiency for all items, except interest in improving or learning about EBP. The difference suggests that SLPs with poor English proficiency have fewer skills than their peers with moderate, good or excellent English proficiency. There were no differences related to time since graduation.

### Opinion

In the opinion of most participants, the application of EBP is necessary for speech-language practice (32.8% agree and 59.8% strongly agree) and considers it necessary to increase the use of scientific evidence in their daily practice (42.6% agree and 36.1% strongly agree). However, most disagree or have doubts that such a practice will positively influence their financial return or that there is no strong scientific evidence for the interventions used (Table 3).

The inferential analysis indicated differences according to English proficiency for the item “the incorporation of EBP places too much responsibility on the speech-language pathologist” (p=0.009). This difference suggests that SLPs with poor English proficiency believe that EBP burdens the professional more frequently than SLPs with greater English proficiency. The time since graduation had a difference for the item “strong scientific evidence is lacking for most of the interventions I use in patients” (p=0.046). In this case, the difference suggests that SLPs who have been trained for at least 10 years agree more often about the lack of evidence than their peers who have been trained for less time.

### Barriers

Regarding barriers, the minority of participants indicated agreement with the item “*The time available is insufficient for the execution of the EBP*” (23.0% agree and 4.9% strongly agree). However, most agree with the items “*There is a lack of articles that make it possible to generalize the findings of the scientific literature to my patient population*” (41.0% agree and 14.8% strongly agree), “*There is difficulty in applying the results of scientific research to patients with unique characteristics*” (43.4% agree and 13.1% strongly agree) and “*There is a lack of collective support among my co-workers for the implementation of EBP*” (37.7% agree and 10.7% strongly agree), as shown in Table 3.

The inferential analysis indicated differences according to the time since graduation only in the item “*The time available is insufficient for the execution of the EBP*” (p=0.036). This difference suggests that the management of time to perform EBP is considered a major barrier for SLPs that have graduated for less than nine years. There were no differences related to English proficiency.

## DISCUSSION

This study sought to characterize the knowledge, skills, opinions, and main barriers related to EBP of Brazilians SLPs in child language.

The first aspect considered relates to continuing education. SLPs have shown to recognize the importance of reading articles and participating in events for scientific updating. By itself, the habit of reading scientific articles indicates a positive attitude towards the pillar of the search for external evidence. However, while more than half of the participants claim to read scientific articles frequently, only a third of SLPs have training in search strategies. This discrepancy may influence the choice of databases used, indicating that there is an ease in performing searches in more commonly disseminated databases and with a greater presence of material in Portuguese, such as Scielo and even Google Scholar. Added to this, the trend of using studies available in full text and virtually, enables the search in more popular journals, a scenario known as FUTON bias (Full Text On the Net)^(14)^.

Knowledge about the basis of EBP does not seem to be part of the academic training of all SLPs. The more recently graduated professionals were less sure about having learned this topic and those with lower English proficiency had less knowledge about EBP. This finding warns the need to reconsider its approach both in undergraduate and graduate studies, since insufficient exposure to EBP decreases the frequency of use of studies for clinical decision-making^(4,5)^.

The aspects with the lowest agreement rate are related to how EBP deals with the professional's limitations and the client's preferences. These aspects make up the EBP triad and should be considered at the time of clinical decision-making^(1,2,15)^.

With regard to skills, most participants indicated using EBP, having an interest in learning or improving their skills, and recognizing the benefits of EBP for decision-making and intervention quality. However, regarding formal training in critical evaluation of scientific articles and interpretation of statistical analysis, most SLPs disagreed or were neutral to the statement.

This difficulty may impair the applicability of the results found in scientific articles to clinical practice, since the critical analysis of the professional is necessary^(1,2,15)^. If we also consider that difficulties in mastering English accentuate the impairment of these skills, we may wonder if even when looking for evidence, these SLPs would not be restricting themselves to national articles. If one of the principles of EBP suggests basing it on the best available scientific evidence^(1)^, it is to be expected that articles published in journals with greater impact should be studied. Without any demerit to national journals, it is necessary to consider that intervention studies are costly and that few are conducted in Brazil, due to the difficulties of investment in the area.

Here it is interesting to point out that in the opinion of the majority there is a lack of strong evidence for the interventions used. However, we note that access to databases such as Cochrane, the ASHA evidence map and SpeechBite is underreported. This factor can be explained both by their lack of knowledge and by the difficulty in accessing and interpreting results in English. It is worth noting, however, that in the context of language, studies that consider the particularities of language are also essential. Therefore, such a finding strengthens that a potentially important barrier to the adoption of evidence is its availability in multiple languages^(9)^.

Regarding the barriers, the scarcity of literature and the difficulty of applying its results in clinical practice are pointed out. Such a pattern is similar to Physical Therapy^(12,13)^, suggesting that the profile identified in our study is similar to other health professions in Brazil. On the other hand, it is necessary to consider that child language still lacks studies with scientific evidence for intervention^(16)^. This alerts us that for the advancement of EBP in the area, researchers' efforts are also necessary, in order to develop and publish studies aimed at clinical practice in childhood.

In addition, the lack of collective support in the workplace is similarly pointed out as a barrier. This finding indicates that the scenario in which the professional is working may be the source of the perceived barriers and limitations to the successful implementation of EBP^(4,11)^. Considering that there is little quality evidence available to support decision-making, it would be beneficial to have spaces for dialogue and exchange among SLPs. This practice could even be implemented from graduation.

This study differs from the literature by not pointing out time as the main barrier to the implementation of EBP^(5,11-13)^. This finding may be associated with the fragility of knowledge about EBP of the professionals surveyed. As pointed out, it is possible that most are still experiencing difficulties in finding scientific evidence, analyzing it critically and considering how to incorporate it into their practice.

In summary, in the opinion of most of the SLPs surveyed, EBP is necessary for speech-language pathology practice and the use of scientific evidence in their daily practice should be greater. Although there are weaknesses in its knowledge and barriers to its implementation, the incorporation of the theme in academic training could strengthen the use of EBP pillars for clinical decision-making^(4,5,7)^.

Among its limitations, the study was developed exclusively in a virtual environment, which may have contributed to the restriction in the number of responses. However, since there is no official record of SLPs working in the area, it was not possible to locate this audience in any other way. Another aspect that could be improved is the data collection instrument itself, since it would be interesting to include open questions that would allow a better understanding of the application of EBP by these professionals.

However, it is worth noting that this study is a pioneer in Brazil in seeking to understand the relationship of SLPs working in child language with EBP. Its results make an important alert to university professors working in undergraduate and graduate studies, as well as indicate to SLPs an essential aspect for their professional improvement.

Thus, this study alerts the academic community to the urgency of considering EBP in the context of Brazilian Speech-Language Pathology. Reducing the distance between training and clinical practice, favoring the use of quality evidence, should be a collective effort of clinicians, professors, and researchers in the area.

## CONCLUSION

Although most of the SLPs surveyed claim to have learned the basics of EBP in their academic training, use and have an interest in improving their skills, there are weaknesses in their knowledge and lack of mastery of search strategies and critical evaluation of scientific articles. Although most agree that EBP’s use is necessary for speech-language pathology practice and consider the need to increase the use of scientific evidence in their daily practice, the lack of articles, difficulties related to the practical application of scientific results and lack of collective support among colleagues are identified as barriers.
