# Supplementary material for: Evidence-based practice and clinical practice in child language: an online survey with Brazilian speech-language pathologists
Source: Codas. 2023 Nov 10;36(1):e20220272. doi: 10.1590/2317-1782/20232022272en (PMC10715581; doi:10.1590/2317-1782/20232022272en)
Supplement: Translation

# Translation: Prática baseada em evidências e atuação clínica em linguagem infantil: uma pesquisa online com fonoaudiólogos brasileiros

**DOI:** 10.1590/2317-1782/20232022272pt

**Authors:** Marcelle Stella de Lima Souza, Ana Manhani Cáceres-Assenço

**Affiliations:** 1 Laboratório de Desenvolvimento da Linguagem, Departamento de Fonoaudiologia, Centro de Ciências da Saúde, Universidade Federal do Rio Grande do Norte - UFRN - Natal (RN), Brasil.

**Keywords:** Linguagem Infantil, Prática Clínica Baseada em Evidências, Terapia da Linguagem, Reabilitação dos Transtornos da Fala e da Linguagem

## Abstract

**Objetivo:**

Caracterizar o conhecimento, habilidades, opiniões e principais barreiras percebidas por fonoaudiólogos, da área de linguagem infantil no Brasil, a respeito da prática baseada em evidências (PBE).

**Método:**

O estudo foi conduzido entre agosto de 2021 e julho de 2022 por meio de um questionário online. Além de dados sociodemográficos e de campo de trabalho, foram considerados 22 itens relacionados à PBE e subdivididos em “conhecimento”, “habilidades”, “opinião” e “barreiras”. Cada item apresentava cinco opções de resposta (discordo totalmente, discordo, não estou decidido, concordo, concordo totalmente). Ao total 122 fonoaudiólogos que atuam na área da linguagem infantil responderam ao questionário. Suas respostas foram descritas pela porcentagem de distribuição de frequência. O tempo de formação e o nível de domínio do inglês foram considerados para comparar o padrão de distribuição das respostas.

**Resultados:**

Apesar da maioria dos fonoaudiólogos reportar ter aprendido as bases da PBE em sua formação acadêmica, há fragilidades em seu conhecimento e falta de domínio das estratégias de busca e avaliação crítica dos artigos científicos. Ainda que a maioria concorde que a aplicação da PBE é necessária para a prática fonoaudiológica e considere precisar aumentar o uso de evidências científicas em sua prática diária, são apontadas como barreiras a falta de artigos, dificuldades relacionadas à aplicação prática de resultados científicos e falta de apoio coletivo entre os colegas.

**Conclusão:**

Este estudo alerta a comunidade acadêmica para a urgência de se considerar a PBE no contexto da Fonoaudiologia brasileira.

## INTRODUÇÃO

A prática baseada em evidências (PBE) equivale a um conjunto de critérios para avaliação da evidência científica. O principal objetivo da PBE é reduzir a incerteza do profissional no momento de uma decisão clínica. Ela associa três pilares: a experiência clínica do profissional; as preferências da família e /ou do cliente; e as evidências externas (informações disponíveis na literatura) e internas (dados coletados na avaliação)^(1,2)^.

A importância da PBE vem sendo frequentemente discutida na comunidade médica e científica^(3)^. No entanto, ainda existem muitas barreiras que impedem sua implementação efetiva, sobretudo na Fonoaudiologia^(4-6)^. Em síntese, apesar de possuírem algum embasamento teórico, os fonoaudiólogos que atuam com transtornos da linguagem em cenário internacional reconhecem o tempo insuficiente, a carga de trabalho extensa, a escassez de pesquisas na área, a qualidade das evidências disponíveis e a falta de recursos no ambiente de trabalho como os principais obstáculos para execução da PBE^(4,5,7,8)^.

As evidências científicas parecem não ser determinantes para a seleção de abordagens de intervenção, principalmente na atuação com linguagem infantil. O fator mais considerado para a tomada de decisão é a experiência clínica do fonoaudiólogo. Por mais que a experiência do profissional seja relevante, a eficácia da PBE depende de sua associação às evidências internas e às preferências dos clientes^(9,10)^. Entretanto, é fundamental pontuar que na atuação fonoaudiológica com os transtornos da linguagem estudos com os melhores níveis de evidência ainda são escassos e há déficit no conhecimento dos profissionais referente aos processos de diagnóstico e intervenção fonoaudiológica^(9,11)^.

Em contexto internacional, os fonoaudiólogos que atuam com linguagem, de maneira geral, possuem atitudes positivas e favoráveis à execução da PBE, embora ainda existam barreiras^(4,5,7,8)^. O treinamento formal acerca das bases da PBE na graduação ou durante a formação continuada aparece como um forte preditor para execução de tal prática durante sua atuação clínica^(4,6,12)^.

No entanto, em contexto nacional há ausência de estudos que investiguem tal cenário. Portanto, o objetivo do estudo foi caracterizar o conhecimento, habilidades, opiniões e principais barreiras percebidas por fonoaudiólogos, da área de linguagem infantil no Brasil, a respeito da prática baseada em evidências.

## MÉTODO

Este estudo está vinculado a um projeto mais amplo que busca investigar como fonoaudiólogos brasileiros atuam no diagnóstico e intervenção em linguagem infantil. O projeto foi aprovado pelo Comitê de Ética em Pesquisa (parecer nº 4.878.557). O termo de consentimento livre esclarecido foi apresentado antes do questionário e a participação ocorreu de forma voluntária e anônima. Os participantes foram informados sobre os objetivos do projeto, o tempo estimado para resposta e a forma de contato com as pesquisadoras em caso de dúvidas. As orientações da Comissão Nacional de Ética em Pesquisa (CONEP) para procedimentos em pesquisas em ambiente virtual, publicadas em fevereiro de 2021, foram seguidas visando preservar a proteção, segurança e os direitos dos participantes.

### Materiais e procedimento

A primeira etapa do estudo consistiu na elaboração de um questionário sobre prática baseada em evidências a partir de instrumentos utilizados pela Fisioterapia^(12,13)^. O questionário foi composto por 22 itens subdivididos nas categorias “conhecimento”, “habilidades”, “opinião” e “barreiras”. Em cada item era preciso especificar o nível de concordância por meio de uma escala Likert com cinco opções de resposta (discordo totalmente, discordo, não estou decidido, concordo, concordo totalmente). O questionário foi precedido por questões relacionadas a aspectos sociodemográficos e do campo de trabalho.

O questionário foi disponibilizado em um formulário aberto na plataforma *Google Forms*. Antes do início da coleta de dados, solicitamos a cinco estudantes de graduação que preenchessem o questionário em busca de erros ou inconsistências, visando o aprimoramento da aplicabilidade do instrumento.

O contato com os participantes em potencial ocorreu por mídias sociais. O estudo foi divulgado por e-mail no boletim informativo de pesquisas aos associados da Sociedade Brasileira de Fonoaudiologia (SBFa), por menção no Instagram de influenciadores digitais e por um anúncio patrocinado no Instagram cujo público-alvo eram os fonoaudiólogos com interesse em linguagem infantil. Devido à recente implementação da lei geral de proteção de dados, o Conselho Federal e os Regionais de Fonoaudiologia informaram ser inviável divulgar o estudo por e-mail aos profissionais ativos.

O acesso ao questionário foi disponibilizado por um link encurtado. Todas as questões eram obrigatórias e a ordem de apresentação foi padronizada. O questionário foi distribuído em oito páginas, cada uma com cerca de cinco questões de múltipla escolha. Ao submeter o questionário não era mais possível realizar alterações e uma cópia das respostas foi enviada ao participante. As respostas foram armazenadas automaticamente em planilha do Google.

Devido às características da plataforma *Google Forms* não foi possível calcular as taxas de visualização do questionário. Porém, todos os questionários submetidos indicaram concordar com o estudo e estavam completos. A priori, nenhuma medida foi adotada para prevenir duplicidade de respostas, entretanto o e-mail foi utilizado para eliminar essas ocorrências antes da análise. Nessa amostra tivemos três formulários duplicados e optamos por manter a primeira submissão.

### Participantes

Como critério de inclusão, os participantes deveriam ter graduação em Fonoaudiologia e atuar com quadros de transtornos de linguagem na infância. De acordo com o Conselho Federal de Fonoaudiologia, em junho de 2021 havia 48.391 fonoaudiólogos no Brasil. Desses, 1155 possuem título de especialista em Linguagem. Entretanto, nem todo profissional que atua com linguagem infantil possui título de especialista e nem todo especialista atua com linguagem infantil. Assim, não foi possível ter uma estimativa precisa da população de interesse.

O cálculo amostral foi realizado no software GPower. Para um tamanho de efeito de 0,3 e um poder estatístico de 0,8, a amostra estimada seria de 167 participantes. Com intuito de alcançar essa amostra, a coleta de dados ocorreu entre agosto de 2021 e julho de 2022.

Por fim, participaram deste estudo 122 fonoaudiólogos que declararam atuar clinicamente na área da linguagem infantil no Brasil. O grupo foi composto 96,7% por mulheres, com faixa etária predominante entre 41 e 50 anos (32,8%), formação universitária em instituição privada (53,3%) e há menos de 5 anos (31,1%), com especialização na área (36,1%), conforme Tabela 1.

**Tabela 1:** Distribuição de frequência da caracterização amostral

Características	%
Gênero	
Feminino	96,7
Masculino	3,3
Idade	
20-30	24,6
31-40	29,5
41-50	32,8
51-60	10,7
+61	2,5
Tipo de instituição em que se graduou	
Privada	53,3
Pública	46,7
Tempo de formação	
- 5 anos	31,1
5 a 9 anos	18,0
10 a 14 anos	13,1
15 a 19 anos	9,8
20 a 24 anos	17,2
+24 anos	10,7
Nível de qualificação profissional	
Doutorado	12,3
Especialização lato sensu	36,1
Graduação	23,8
Mestrado acadêmico	19,7
Mestrado profissional	4,1
Pós-doutorado	4,1

### Análise dos dados

O tratamento estatístico dos dados foi realizado no software SPSS versão 24. Para a análise descritiva foi utilizada a distribuição de frequência. A análise inferencial foi realizada usando o teste qui-quadrado considerando para o tempo de formação duas categorias (formado até 9 anos e formado a partir de 10 anos) e para o domínio do inglês três categorias (pobre, moderado, bom ou excelente). O nível de significância adotado foi de 5%. Além do cálculo amostral, o software *GPower* foi utilizado para calcular o tamanho do efeito e o poder estatístico.

## RESULTADOS

Os participantes demonstraram preocupação com a formação continuada, pois 82% afirmam ter participado de congressos científicos, cursos ou atualizações na área (40,2% muito frequentemente, 41,8% frequentemente). Além disso, 63,1% afirmam ter o hábito de ler artigos científicos, sendo 20,5% muito frequentemente e 42,6% frequentemente. As bases de dados mais utilizadas para a busca dos artigos foram Scielo (33,6%), PubMed (24,8%) e Google acadêmico (23,2%). Nenhum participante indicou utilizar a base SpeechBite (Tabela 2).

**Tabela 2:** Distribuição de frequência da busca por formação continuada e evidências externas

Características	%
Formação continuada nos últimos 3 anos	
Muito frequentemente	40,2
Frequentemente	41,8
Ocasionalmente	13,9
Raramente	2,5
Nunca	1,6
Leitura de artigos	
Muito frequentemente	20,5
Frequentemente	42,6
Ocasionalmente	29,5
Raramente	6,6
Nunca	0,8
Base de dados que utiliza	
Scielo	33,6
PubMed	24,8
Google Acadêmico	23,2
Periódicos Capes	8,8
Mapa de evidências da ASHA	6,4
Não se aplica	3,2
Cochrane	0,8
Bireme	0,8
SpeechBite	0,0

### Conhecimento

Com relação ao conhecimento, a maioria dos participantes indicou ter aprendido as bases da PBE durante a formação acadêmica (36,1% concordo e 20,5% concordo totalmente) e considerar que ela melhora a qualidade do atendimento (35,2% concordo e 54,1% concordo totalmente) e auxilia na tomada de decisões sobre o tratamento (47,5% concordo e 39,3% concordo totalmente). Porém inconsistências foram identificadas nos itens “*A PBE não leva em conta as limitações da minha prática clínica*” (45,1% não estou decidido, 19,7% concordo e 3,3% concordo totalmente) e “*A PBE não leva em conta as preferências do paciente*” (29,5% não estou decidido, 15,6% concordo e 2,5% concordo totalmente), conforme Tabela 3.

**Tabela 3:** Barreiras, habilidades, conhecimento e opinião dos fonoaudiólogos sobre a prática baseada em evidências

	Discordo totalmente		Discordo	Não estou decidido	Concordo	Concordo totalmente	X^2^ Domínio Inglês	X^2^ Tempo Formação
**Conhecimento**								
Na minha formação acadêmica aprendi as bases para a PBE		12,3	15,6	15,6	36,1	20,5	<0,001*	0,021*
A PBE melhora a qualidade do atendimento ao paciente		0,0	0,8	9,8	35,2	54,1	0,314	0,059
A PBE não leva em conta as limitações da minha prática clínica		6,6	25,4	45,1	19,7	3,3	0,203	0,290
A PBE não leva em conta as preferências do paciente		10,7	41,8	29,5	15,6	2,5	0,197	0,343
A PBE ajuda a tomar decisões sobre o tratamento dos pacientes		0,8	1,6	10,7	47,5	39,3	0,053	0,584
**Habilidades**								
Faço uso da PBE na prática clínica e no planejamento terapêutico		1,6	2,5	10,7	44,3	41,0	0,018*	0,266
Tenho interesse em aprender ou aprimorar as habilidades necessárias para incorporar PBE em minha prática		0,0	0,0	2,5	37,7	59,8	0,521	0,106
Possuo treinamento em estratégias de busca para encontrar literatura online relevante para a minha prática		9,0	21,3	14,8	30,3	24,6	0,001*	0,181
Possuo treinamento formal em avaliação crítica de artigos científicos como parte de minha preparação acadêmica		15,6	22,1	17,2	19,7	25,4	0,003*	0,172
Sou capaz de fazer uma análise crítica de artigos científicos		0,8	14,8	21,3	38,5	24,6	<0,001*	0,204
Sou capaz de encontrar artigos científicos relevantes para responder às minhas questões clínicas		0,8	8,2	18,0	43,4	29,5	<0,001*	0,421
Sou capaz de compreender a análise estatística dos artigos científicos		4,9	12,3	31,1	31,1	20,5	<0,001*	0,173
**Opinião**								
Na minha perspectiva a aplicação da PBE é necessária para a prática da Fonoaudiologia		0,0	0,0	7,4	32,8	59,8	0,156	0,419
Preciso aumentar o uso de evidências científicas em minha prática diária.		1,6	12,3	7,4	42,6	36,1	0,348	0,103
Meu ganho financeiro irá aumentar se eu incorporar a PBE na minha prática		11,5	23,0	33,6	26,2	5,7	0,872	0,285
Estão faltando fortes evidências científicas para a maioria das intervenções que eu uso nos pacientes		8,2	35,2	28,7	22,1	5,7	0,239	0,046
A incorporação da PBE coloca uma responsabilidade demasiada sobre o fonoaudiólogo		14,8	34,4	26,2	19,7	4,9	0,009*	0,097
**Barreiras**								
O local em que trabalho incentiva o uso dos resultados de investigações atuais na prática clínica		10,7	14,8	15,6	30,3	28,7	0,220	0,290
O tempo disponível é insuficiente para a execução da PBE		5,7	31,1	35,2	23,0	4,9	0,537	0,036
Há falta de artigos que possibilitem a generalização dos achados da literatura científica para a minha população de pacientes		2,5	20,5	21,3	41,0	14,8	0,469	0,399
Há dificuldade na aplicação dos resultados da investigação científica para pacientes com características únicas		0,8	16,4	26,2	43,4	13,1	0,653	0,679
Há falta de apoio coletivo entre meus colegas de trabalho para a execução da PBE		5,7	20,5	25,4	37,7	10,7	0,747	0,880

* Diferença estatística p<0,05 - teste Qui-quadrado (X^2^)

A análise inferencial indicou diferenças de acordo com o domínio do inglês (p<0,001) e tempo de formação (p=0,021) apenas no item “*Na minha formação acadêmica aprendi as bases para a PBE*”. Com relação ao domínio do inglês, a diferença sugere que fonoaudiólogos com domínio pobre possuem menos conhecimento sobre PBE do que aqueles com domínio moderado ou bom e excelente. Com relação ao tempo de formação, a diferença sugere que os fonoaudiólogos formados até 9 anos estavam menos decididos sobre terem tido acesso a esse conteúdo do que seus pares formados há mais de 10 anos.

### Habilidades

No que se refere às habilidades, a maioria dos participantes indicou utilizar a PBE (44,3% concordo e 41,0% concordo totalmente) e ter interesse em aprender ou aprimorar suas habilidades (37,3% concordo e 59,8% concordo totalmente). Porém, quando se trata de treinamento formal em avaliação crítica de artigos científicos, a maioria discorda ou se mostra neutra (15,6% discordo, 22,1% discordo totalmente, 17,2% não estou decidido).

24,6% dos fonoaudiólogos indicam possuir treinamento em estratégias de busca de artigos científicos, ao passo que 22,1% deles discordou de tal afirmação. Ademais, 31,1% se consideram capazes de compreender a análise estatística dos artigos, mas outros 31,1% se mostraram indecisos sobre esse item, conforme Tabela 3.

A análise inferencial indicou diferenças de acordo com o domínio do inglês para todos os itens, exceto o interesse em aprimorar ou aprender sobre PBE. A diferença sugere que os fonoaudiólogos com domínio pobre do inglês possuem menos habilidades do que seus pares com domínio moderado, bom ou excelente. Não houve diferenças relacionadas ao tempo de formação.

### Opinião

Na opinião da maioria dos participantes a aplicação da PBE é necessária para a prática fonoaudiológica (32,8% concordo e 59,8% concordo totalmente) e considera preciso aumentar o uso de evidências científicas em sua prática diária (42,6% concordo e 36,1% concordo totalmente). No entanto, a maioria discorda ou tem dúvidas de que tal prática irá influenciar positivamente em seu retorno financeiro ou que não haja evidências científicas fortes para as intervenções que utiliza (Tabela 3).

A análise inferencial indicou diferenças de acordo com o domínio do inglês para o item “A incorporação da PBE coloca uma responsabilidade demasiada sobre o fonoaudiólogo” (p=0,009). Essa diferença sugere que fonoaudiólogos com pobre domínio do inglês acreditam que a PBE sobrecarrega o profissional com maior frequência do que fonoaudiólogos com maior domínio do inglês. O tempo de formação teve diferença para o item “Estão faltando fortes evidências científicas para a maioria das intervenções que eu uso nos pacientes” (p=0,046). Nesse caso, a diferença sugere que os fonoaudiólogos formados há pelo menos 10 anos concordam com mais frequência sobre a falta de evidências do que seus pares formados há menos tempo.

### Barreiras

Com relação às barreiras, a minoria dos participantes indicou concordar com o item “*O tempo disponível é insuficiente para a execução da PBE*” (23,0% concordo e 4,9% concordo totalmente). Porém, a maioria concorda com os itens “*Há falta de artigos que possibilitem a generalização dos achados da literatura científica para a minha população de pacientes*” (41,0% concordo e 14,8% concordo totalmente), “*Há dificuldade na aplicação dos resultados da investigação científica para pacientes com características únicas*” (43,4% concordo e 13,1% concordo totalmente) e “*Há falta de apoio coletivo entre meus colegas de trabalho para a execução da PBE*” (37,7% concordo e 10,7% concordo totalmente), conforme Tabela 3.

A análise inferencial indicou diferenças de acordo com o tempo de formação apenas no item “*O tempo disponível é insuficiente para a execução da PBE*” (p=0,036). Essa diferença sugere que o gerenciamento do tempo para execução da PBE é considerado uma barreira maior para os fonoaudiólogos formados há menos de nove anos. Não houve diferenças relacionadas ao domínio do inglês.

## DISCUSSÃO

Este estudo buscou caracterizar o conhecimento, habilidades, opiniões e principais barreiras relacionadas à PBE de fonoaudiólogos na área de linguagem infantil no Brasil.

O primeiro aspecto considerado se relaciona à formação continuada. Os fonoaudiólogos demonstraram reconhecer a importância de ler artigos e participar de eventos para atualização científica. Por si só, o hábito de leitura de artigos científicos indica atitude positiva em relação ao pilar da busca por evidências externas. Porém, enquanto mais da metade dos participantes afirma ler artigos científicos com frequência, apenas um terço dos fonoaudiólogos possui treinamento em estratégias de busca. Tal discrepância pode influenciar na escolha das bases de dados utilizadas, indicando que há uma facilidade em realizar buscas em bases mais comumente disseminadas e com maior presença de material em português, como Scielo e até mesmo o Google Acadêmico. Somado a isso, a tendência da utilização de estudos disponibilizados na íntegra e virtualmente, viabiliza a busca em periódicos mais populares, cenário conhecido como viés de FUTON (Full Text On the Net)^(14)^.

O conhecimento sobre as bases da PBE parece não fazer parte da formação acadêmica de todos os fonoaudiólogos. Os profissionais formados mais recentemente tinham menos certeza sobre terem aprendido esse tema e aqueles com menor domínio do inglês possuem menos conhecimento sobre PBE. Tal achado alerta para a necessidade de se reconsiderar sua abordagem tanto na graduação, quanto na pós-graduação, já que a exposição insuficiente à PBE diminui a frequência de uso de estudos para tomada de decisão clínica^(4,5)^.

Os aspectos com menor índice de concordância se relacionam a como a PBE lida com as limitações do profissional e as preferências do cliente. Esses aspectos compõem a tríade da PBE e devem ser considerados no momento de tomada de decisão clínica^(1,2,15)^.

No que se refere às habilidades, a maioria dos participantes indicou utilizar a PBE, ter interesse em aprender ou aprimorar suas habilidades, além de reconhecer os benefícios da PBE para a tomada de decisões e qualidade da intervenção. Porém, com relação ao treinamento formal em avaliação crítica de artigos científicos e interpretação da análise estatística, a maioria dos fonoaudiólogos discordou possuir ou se mostrou neutra à afirmação.

Essa dificuldade pode prejudicar a aplicabilidade dos resultados encontrados em artigos científicos à prática clínica, já que a análise crítica do profissional é necessária^(1,2,15)^. Se considerarmos ainda que dificuldades no domínio do inglês acentuam o comprometimento dessas habilidades, podemos nos questionar se mesmo ao buscarem evidências, esses fonoaudiólogos não estariam se restringindo a artigos nacionais. Se um dos princípios da PBE sugere o embasamento nas melhores evidências científicas disponíveis^(1)^, há de se esperar que artigos publicados em revistas com maior impacto devam ser estudados. Sem nenhum demérito aos periódicos nacionais, é preciso considerar que estudos de intervenção são onerosos e que poucos são conduzidos no Brasil, devido às dificuldades de investimento na área.

Aqui é interessante pontuar que na opinião da maioria há falta de fortes evidências para as intervenções utilizadas. Todavia, notamos que o acesso a bases de dados como a Cochrane, o mapa de evidências da ASHA e a SpeechBite é pouco reportado. Esse fator pode tanto ser explicado por seu desconhecimento, quanto pela dificuldade em acessar e interpretar resultados em língua inglesa. Vale destacar, porém que no contexto da linguagem, estudos que considerem as particularidades da língua também são essenciais. Portanto, tal achado fortalece que uma barreira potencialmente importante para a adoção de evidências é sua disponibilidade em vários idiomas^(9)^.

Com relação às barreiras, a escassez de literatura e a dificuldade de aplicar seus resultados na prática clínica são apontadas. Tal padrão é semelhante ao de fisioterapeutas^(12,13)^, sugerindo que o perfil identificado em nosso estudo seja similar ao de outras profissões da saúde no Brasil. Por outro lado, é necessário considerar que a área de linguagem infantil ainda carece de estudos com evidências científicas para intervenção^(16)^. O que nos alerta que para o avanço da PBE na área são necessários esforços também dos pesquisadores, no sentido de desenvolver e publicar estudos voltados para a prática clínica na infância.

Além disso, a falta de apoio coletivo no ambiente de trabalho é similarmente apontada como uma barreira. Esse achado indica que o cenário em que o profissional está atuando pode ser fonte das barreiras e limitações percebidas para o sucesso da implementação da PBE^(4,11)^. Ao considerar que há poucas evidências de qualidade disponíveis para embasar a tomada de decisão, seria benéfico haver espaços de diálogo e troca entre os fonoaudiólogos. Essa prática inclusive poderia ser implementada desde a graduação.

Este estudo destoa da literatura ao não apontar o tempo como a principal barreira para implementação da PBE^(5,11-13)^. Esse achado pode estar associado à fragilidade dos conhecimentos sobre PBE dos profissionais inquiridos. Como apontado, é possível que a maioria ainda esteja enfrentando dificuldades para encontrar evidências científicas, analisá-las de forma crítica e considerar como incorporá-las em sua prática.

Em síntese, na opinião da maioria dos fonoaudiólogos inquiridos, a PBE é necessária para a prática fonoaudiológica e o uso de evidências científicas em sua prática diária deveria ser maior. Por mais que haja fragilidades em seu conhecimento e barreiras para sua implementação, a incorporação do tema na formação acadêmica poderia fortalecer o uso dos pilares da PBE para tomada de decisão clínica^(4,5,7)^.

Dentre suas limitações, o estudo foi desenvolvido exclusivamente em meio virtual, o que pode ter contribuído para a restrição no número de respostas. Porém, uma vez que não há um registro oficial dos fonoaudiólogos que atuam na área não foi possível localizar esse público de outra maneira. Outro aspecto que poderia ser aprimorado é o próprio instrumento de coleta de dados, visto que seria interessante incluir questões abertas que permitissem ter uma melhor compreensão da aplicação da PBE por esses profissionais.

Entretanto, vale destacar que este estudo é pioneiro no Brasil ao buscar compreender a relação de fonoaudiólogos que atuam na área de linguagem infantil com a PBE. Seus resultados fazem um importante alerta aos docentes atuantes na graduação e na pós-graduação, bem como indicam aos fonoaudiólogos um aspecto essencial para seu aprimoramento profissional.

Assim, este estudo alerta a comunidade acadêmica para a urgência de se considerar a PBE no contexto da Fonoaudiologia brasileira. Reduzir a distância entre a formação e a prática clínica, favorecendo o uso de evidências de qualidade, deve ser um esforço coletivo de clínicos, docentes e pesquisadores da área.

## CONCLUSÃO

Embora a maioria dos fonoaudiólogos inquiridos afirme ter aprendido as bases da PBE em sua formação acadêmica, utilizar e ter interesse em aprimorar suas habilidades, há fragilidades em seu conhecimento e falta de domínio das estratégias de busca e avaliação crítica dos artigos científicos. Ainda que a maioria concorde que a aplicação da PBE é necessária para a prática fonoaudiológica e considere precisar aumentar o uso de evidências científicas em sua prática diária, são apontadas como barreiras a falta de artigos, dificuldades relacionadas à aplicação prática de resultados científicos e falta de apoio coletivo entre os colegas.
